# Predictive model and risk analysis for coronary heart disease in people living with HIV using machine learning

**DOI:** 10.1186/s12911-024-02511-5

**Published:** 2024-04-25

**Authors:** Zengjing Liu, Zhihao Meng, Di Wei, Yuan Qin, Yu Lv, Luman Xie, Hong Qiu, Bo Xie, Lanxiang Li, Xihua Wei, Die Zhang, Boying Liang, Wen Li, Shanfang Qin, Tengyue Yan, Qiuxia Meng, Huilin Wei, Guiyang Jiang, Lingsong Su, Nili Jiang, Kai Zhang, Jiannan Lv, Yanling Hu

**Affiliations:** 1 Information and Management College of Guangxi Medical University, Nanning, Guangxi 530021 China; 2 Guangxi Clinical Center for AIDS Prevention and Treatment, Chest Hospital of Guangxi Zhuang Autonomous Region, No. 8 Yangjiaoshan Road, Liuzhou, Guangxi 545005 China; 3 Life Sciences College of Guangxi Medical University, Nanning, Guangxi 530021 China; 4 Basic Medical College of Guangxi Medical University, Nanning, Guangxi 530021 China; 5 Collaborative Innovation Centre of Regenerative Medicine and Medical BioResource Development and Application Co- constructed by the Province, Ministry of Guangxi Medical University, Nanning, Guangxi 530021 China; 6 Department of rehabilitation medicine, Department of the First affliated hospital of Guangxi Medical University, Nanning, Guangxi 530021 China; 7 Affiliate Hospital of Youjiang Medical University for Nationalities, Baise, Guangxi 533000 China; 8 Faculty of Data science, City University of Macau, 999078 Macau, China

**Keywords:** Human immunodeficiency virus (HIV), Coronary heart disease (CHD), Machine learning, Risk assessment, Electronic medical record (EMR)

## Abstract

**Objective:**

This study aimed to construct a coronary heart disease (CHD) risk-prediction model in people living with human immunodeficiency virus (PLHIV) with the help of machine learning (ML) per electronic medical records (EMRs).

**Methods:**

Sixty-one medical characteristics (including demography information, laboratory measurements, and complicating disease) readily available from EMRs were retained for clinical analysis. These characteristics further aided the development of prediction models by using seven ML algorithms [light gradient-boosting machine (LightGBM), support vector machine (SVM), eXtreme gradient boosting (XGBoost), adaptive boosting (AdaBoost), decision tree, multilayer perceptron (MLP), and logistic regression]. The performance of this model was assessed using the area under the receiver operating characteristic curve (AUC). Shapley additive explanation (SHAP) was further applied to interpret the findings of the best-performing model.

**Results:**

The LightGBM model exhibited the highest AUC (0.849; 95% CI, 0.814–0.883). Additionally, the SHAP plot per the LightGBM depicted that age, heart failure, hypertension, glucose, serum creatinine, indirect bilirubin, serum uric acid, and amylase can help identify PLHIV who were at a high or low risk of developing CHD.

**Conclusion:**

This study developed a CHD risk prediction model for PLHIV utilizing ML techniques and EMR data. The LightGBM model exhibited improved comprehensive performance and thus had higher reliability in assessing the risk predictors of CHD. Hence, it can potentially facilitate the development of clinical management techniques for PLHIV care in the era of EMRs.

## Introduction

Coronary atherosclerotic heart disease (coronary heart disease, CHD) is currently the most common cardiovascular disease in the world. The incidence and mortality rates of cardiovascular diseases, particularly CHD, in people living with HIV (PLHIV) have been increasing annually. [1]. The mortality rate of myocardial infarction is 1.5 to 1.7 times that of the general population, and the average age of death is about 48 years old, which is far lower than that of the general population [2]. Although PLHIV have a very high awareness of traditional risk factors for CHD, the incidence of CHD has not been reduced in this group, which may be related to the particularity of PLHIV themselves. Compared with the general population, the age of onset of CHD in PLHIV is about 10 years earlier. With increased age, the risk of CHD increases yearly. Many and complex traditional risk factors influence CHD, including male gender, smoking, and high-density lipoprotein Lowered protein cholesterol are high-risk factors [3]. Therefore, analyzing the changes in clinical characteristics of PLHIV with CHD and exploring the risk factors for patients with comorbidities has great significance in the disease prevention and treatment of this special group.

The death rate of AIDS has gradually decreased owing to extended application of highly active antiretroviral therapy (HAART). In turn, the life expectancy of PLHIV has been prolonged even in less-developed areas like sub-Saharan Africa [4, 5]. However, although AIDS has transformed into a manageable chronic disease [6], the risk of basic diseases such as cardiovascular disease (CVD) has increased. According to a meta-analysis research, the risk of CVD among PLHIV is 2.16 times than that in the general population [7]. The mortality rate among people living with HIV (PLHIV) is 1.6 per 1000 people and has been observed to increase annually [8]. This rise in mortality is primarily attributed to the long-term effects of antiretroviral therapy, which include hypercoagulability, co-infection, and immune activation, as identified in studies [9–12]. Protease inhibitor therapy can also cause side effects like hyperlipidemia and insulin resistance, which further promote the pathogenesis of CVD [13]. Given that CVD has become the first cause of non-AIDS death among PLHIV, the management of CVD in PLHIV should be given focus to control the death rate.

Risk factors help predict potential negative events in advance. In PLHIV, the risk factors are similar but more severe than traditional CVD. For example, the prevalence of hypertension, diabetes, atherosclerosis, and dyslipidemia is significantly higher than in the non-HIV-infected population [14, 15]. Unfortunately, few studies have reported on risk factors in Chinese PLHIV with CHD. Only a retrospective research about that risk factors is available. Its results show that CHD does not change in HIV-positive patients, except for the body mass index being lower than that in HIV-negative patients. Most of the clinical characteristics of HIV-positive patients with CHD are similar to those of HIV-negative ones. However, the levels of total cholesterol, high-density lipoprotein cholesterol, and low-density lipoprotein cholesterol in HIV-positive patients are significantly lower, the heart is significantly enlarged, and the incidence of acute coronary syndrome is reduced [16]. Specifically, males and young people infected with HIV are more likely to smoke than the non-HIV group, which is the most important risk factor for acute coronary syndrome [17–19]. However, a 6-year follow-up study has revealed that in a population without CVD risk factors, the probability of acute myocardial infarction of PLHIV is twice that of non-infected ones. The former group of PLHIV are 7 to 10 times more likely to have an acute myocardial infarction than those without HIV. Even after controlling for traditional CVD risk factors, people with HIV are twice as likely to develop CVD as those without HIV [20]. The reason may be that most PLHIV do not have high risk factors for conventional CVD at the time of diagnosis. Current risk factors fail to assist predicting potential CVD risk in PLHIV, so a more accurate predictive indicator is well positioned to be discovered. Yet, traditional CVD risk-assessment tools may underestimate the CVD risk of PLHIV.

Machine learning (ML) algorithms exhibit improved discrimination capacity and generalizability in high-dimensional data, indicating that they are not confined by strict exclusion and inclusion criteria. Thus, the actual health status of individuals are available to these algorithms [21]. This method addresses limitations in existing risk-prediction techniques. ML models, leveraging electronic medical records (EMRs), can enhance clinical diagnostic accuracy and decision by physicians [22]. Therefore, multiple ML algorithms have been extensively used to predict CVD [23, 24], including prediction of 3-year all-cause mortality in patients with heart failure caused by CHD [25], and the classification of in-hospital mortality in chronic kidney disease patients with coronary artery disease [26]. ML algorithms can be useful in the identification of patients with CVD. Often, many elements contribute to classifying patients who are at risk for these common diseases. ML methods can help identify hidden patterns in these factors that may otherwise be missed. Moreover, no predictive models of CHD in PLHIV based on EMRs have been constructed yet using ML.

Accordingly, the present study aimed to determine accurate the predictive risk factors for CHD in PLHIV by establishing a risk-prediction model based on ML. We compared the predictive performance of seven ML algorithms in detail, selected the model with the best comprehensive performance, and visually explained the model. This model can assist clinicians to screen HIV patients who may experience CHD in the future, discovering the risk factors for CHD among HIV-infected patients and provide evidence-based guidance for the prevention of CHD among HIV-infected patients in the Chinese population.

## Materials and methods

### Data source

Data were acquired from the EMR database of inpatients of Guangxi Chest Hospital, a unique provincial clinical center for the prevention and control of HIV/AIDS. The EMR database was linked to collect demographic information (e.g., age and gender), clinical laboratory measurement records, and clinical diagnoses of inpatients. Valid and intact patient data obtained between June 2016 and October 2021 were included in the study. Furthermore, to maintain privacy, identity-related information of all individuals was concealed during data acquisition.

### Study population

Individuals were diagnosed with HIV, per the International Classification of Diseases (ICD)-10 codes. The inclusion criteria were as follows: (1) age above 18 years; (2) patient was confirmed to be HIV-infected according to *the Guidelines for the Diagnosis and Treatment of AIDS in China (2018 Edition)*; and (3) results of biochemical examination during hospitalization can be queried. Meanwhile, patients with incomplete medical histories were excluded.

### Data imputation

To enhance data utilization, variables exhibiting more than 20% missing data were excluded before performing data interpolation. For others, the missing data were imputed with the help of the random forest (RF) method and algorithms, which are great for imputing missing data. They are desirable because they can handle mixed types of missing data. Additionally, they are adaptive to nonlinearity and interactions and can potentially be scaled to big-data settings [27].

### Class-imbalance problem

The ML classifier is generally more biased toward the majority class when dealing with datasets having a class imbalance, thereby leading to bad classification for the minority class. In the case of such issues, the majority is labeled as a single class, whereas the minority is labeled as the other class [28]. In this dataset, CHD individuals accounted for 3.53% of PLHIV. Furthermore, an imbalanced distribution of these two classes was observed, potentially leading to subpar prediction performance of the minority class in the prediction model [28]. A cost-sensitive learning method, used in data mining, aims to produce accurate results for class-imbalanced datasets with minimal cost by re-weighting the cost matrix, allowing the classifier to focus on fewer weight cases and avoid predicting high-cost cases [29].

### Model development and evaluation

Seven ML algorithms implemented in the Python package 3.10.9 were as follows: a light gradient-boosting machine (LightGBM), lasso-logistic regression, eXtreme gradient boosting (XGBoost), adaptive boosting (AdaBoost), decision tree, multilayer perceptron (MLP), and support vector machine (SVM). They aided the identification of the most informative variables for CHD risk prediction in PLHIV, as well as the development of models that predicted CHD in PLHIV as a binary outcome (absence or presence), per the laboratory and clinical diagnosis values of the chosen predictor variables.

LightGBM and XGBoost are members of the boosting algorithm family and utilize the negative gradient of the loss function to compute the residual and ascertain the ideal solution. LightGBM is a highly efficient and accurate implementation of the gradient-boosting decision tree (GBDT). Additionally, compared with XGBoost, LightGBM trains faster, consumes less memory, has higher accuracy, and can handle larger amounts of data [30]. Meanwhile, AdaBoost algorithm [31] is a classic Boosting algorithm that trains various classifiers (weak classifiers) for the same training set. Subsequently, they are assembled to create a stronger final classifier (strong classifier). An enhancement of the logistic regression method, MLP [32] is a feedforward artificial neural network model that maps several datasets of inputs onto datasets of a single output. Moreover, SVM [33] is a binary classification model that maps data in a high-dimensional space. It also finds a hyperplane in the space to maximize the distance among various data points and the hyperplane, which distinguishes it from MLP. Furthermore, the decision-tree [34] learning method constitutes a non-parametric supervised approach. It summarizes decision rules from a series of data with labels and features and subsequently illustrates them as a tree graph to resolve regression and classification issues. Then, logistic regression converts the output results of linear regression into probability values via a function to realize sample classifications. Lasso regression was used to screen features and eliminate the multicollinearity among them.

Data were segregated randomly into training and validation datasets with the help of the Python package (Scikit-learn). Among these data, 80% aided model training, whereas the remaining 20% helped validate its predictive performance. In this study, sensitivity, accuracy, specificity, negative predictive value, positive predictive value (PPV), the areas under the receiver operator characteristic curves (ROC-AUC), and F1 score (2*((precision*recall)/(precision + recall)) were used to compare model performance. Moreover, a 10-fold cross-validation was performed to compare the AUC of the seven ML algorithms and to ascertain the overall best performance.

To comprehend the findings of the ML models more coherently, the Shapley additive explanation (SHAP) method aided the visualization analysis. This method was applied to comprehend the findings of the best prediction model in terms of performance. For this purpose, the individual contribution of each variable was computed [35]. A SHAP value denotes the contribution of the feature to the outcome value. A positive value indicates that the feature promotes the likelihood of a positive outcome, whereas a negative one indicates that the feature decreases the likelihood of a positive outcome.

### Statistical analyses

**Fig. 1:**
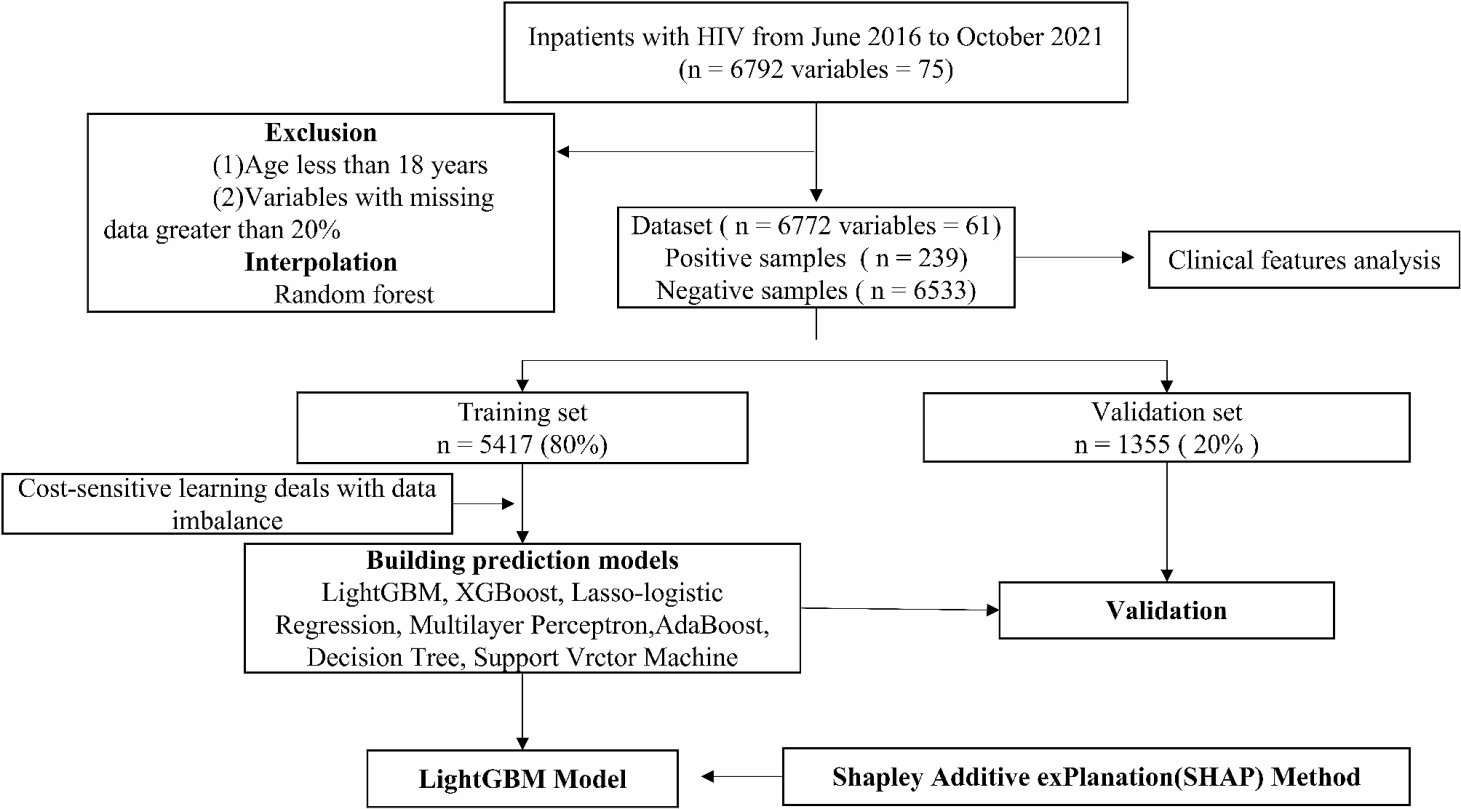
General schema for building and evaluating the prediction model. Positive samples were defined as PLHIV with CHD, whereas negative samples were PLHIV without CHD

Analyses were conducted with the help of SPSS (ver. 26.0) software (IBM, Chicago, IL, USA). The clinical-feature analysis of the complete dataset was performed in the interpolated dataset. The continuous variables were reported as the median (IQR) because the data were non-normally distributed. Meanwhile, the categorical variables were represented as numbers and percentages. Additionally, the continuous and categorical variables were compared with the help of the Wilcoxon rank sum test and the Chi-square test, respectively. For all tests, *P* < 0.05 was deemed statistically significant. The general schema for building this prediction model is also illustrated in Fig. 1.

## Results

From the data of 6792 PLHIV including 239 CHD patients and 6553 non-CHD patients, 75 variables were extracted. Twenty patients younger than 18 years of age were excluded, leaving 6772 patients. Then, 14 variables were removed because more than 20% date were missing, ultimately leaving 61 variables. These variables included demography, laboratory measurements, and diseases besides HIV and CHD (e.g., hypertension). Post-interpolation with RF, clinical-feature analysis of datasets was performed, and the obtained findings are depicted in Table 1. The mean age of the 6772 PLHIV was 54 (IQR: 43–64) years, including 5152 males (76.08%) and 1620 females (23.92%). Additionally, a total of 239 individuals (3.53%) were diagnosed with CHD, among which 82.43% were males and 17.57% were females.

**Table 1:** Demographic and clinical characteristics of the included patients

Variables		All (*n* = 6772)	non-CHD (*n* = 6533)	CHD (*n* = 239)	*P*-Value
Age, years		54(43–64)	53(43–64)	67(60–74)	< 0.001
Sex	Male	5152	4955	197	0.019
	Female	1620	1578	42	
Ethnicity	Han	3327	3199	128	0.12
	Zhuang	2994	2892	102	
	Others	451	422	9	
Marital status	Unmarried	1748	1719	29	< 0.001
	Married	3405	3650	155	
	Divorce /widowhood	1119	1164	55	
Hypertention	Yes	451	391	60	< 0.001
Diabetes	Yes	262	235	27	< 0.001
COPD	Yes	152	131	21	< 0.001
Heart failer	Yes	323	244	79	< 0.001
CD4 count, cell/µL		72(16–215)	69(15–212)	137(35–255)	< 0.001
CD45count, cell/µL		786.25(412-1284.75)	784(407-1280.50)	891(501–1359)	0.022
CD8 count, cell/µL		379(202–639)	379(202–638)	379(232–665)	0.517
D-Dimer, mg/L		1.79(0.6–5.2)	1.81(0.60–5.24)	1.50(0.57–3.78)	0.047
CD3 count, cell/µL		520(259-886.75)	517(256–880)	607(321–958)	0.030
ALB, U/L		32.60(27.37–37.20)	32.60(27.30–37.20)	32.20(27.80–36.70)	0.763
WBC, 10^9/L		4.89(3.31–6.92)	4.87(3.29–6.90)	5.22(3.70–7.08)	0.033
APTT, s		30.20(25-41.40)	30.20(25-41.80)	28.90(23.70–35.70)	0.003
MONO, 10^9/L		0.47(0.31–0.66)	0.47(0.31–0.65)	0.52(0.35–0.70)	0.019
LDL-c, mmol/L		2.01(1.52–2.57)	2.01(1.52–2.56)	2.05(1.60–2.63)	0.156
AMY, U/L		90.77(69–117)	90.75(69-117.41)	91.74(67–112)	0.304
Ca, mmol/L		2.06(1.93–2.17)	2.06(1.93–2.17)	2.05(1.92–2.15)	0.646
TG, mmol/L		1.42(1.04–2.02)	1.42(1.04–2.03)	1.34(1.03–1.84)	0.057
HDL-c, mmol/L		0.85(0.60–1.11)	0.84(0.59–1.11)	0.91(0.69–1.13)	0.006
GGT, U/L		63(35–121)	63(35–121)	61(33–121)	0.508
ALT, U/L		22(13–37)	22(13–37)	20(14–37)	0.842
AST/ALT		1.38(0.94–2.09)	1.39(0.93–2.09)	1.33(1-1.93)	0.847
AST, U/L		27(20–44)	27(20–44)	27(20–46)	0.721
Cys-C, mg/L		1.17(0.98–1.54)	1.17(0.98–1.54)	1.27(1.01–1.72)	0.010
RBC, 10^12/L		3.41(2.77–4.02)	3.40(2.77–4.02)	3.47(2.79–4.03)	0.554
RDW-CV		15(13.97-18)	15.25(13.96-18)	15(14–17)	0.031
RDW-SD		49(44–57)	49(44–57)	49(44–55)	0.369
HCT		31(25.70–35.50)	30.90(25.70–35.50)	31.80(26.60–35.80)	0.217
Scr, µmol/L		70(58–88)	70(57–88)	78(66–106)	< 0.001
Ccr, mL/min		64.36(47.86–77.67)	64.36(47.86–77.67)	58.95(42.40-75.23)	0.010
CK, U/L		65(39-110.90)	65(39-110.66)	67.70(42–115)	0.169
K, mmol/L		3.7(3.30–4.10)	3.70(3.30–4.10)	3.70(3.40–4.18)	0.143
I-Bil, µmol/L		3.72(2.50–5.79)	3.73(2.50–5.80)	3.59(2.50–5.43)	0.375
LYM, 10^9/L		0.98(0.56–1.53)	0.97(0.56–1.52)	1.13(0.67–1.56)	0.015
Cl, mmol/L		102.90(100–106)	102.90(100–106)	102.90(100–106)	0.274
Mg, mmol/L		0.78(0.71–0.87)	0.78(0.71–0.86)	0.78(0.71–0.85)	0.788
Na, mmol/L		137(133–139)	137(133–139)	136(132–140)	0.377
UREA, mmol/L		4.15(3.10–5.90)	4.10(3.10–5.90)	4.70(3.60–6.90)	< 0.001
SUA, µmol/L		300(221–398)	300(221–396)	319(233–443)	0.005
TT, s		19.60(18.20–22.30)	19.60(18.20–22.30)	19.60(18.30–21.60)	0.566
PT, s		12.20(11.10–15)	12.20(11.10–15.10)	11.90(10.80–13.60)	0.021
MCV, fL		90.10(83.70–96.40)	90(83.70–96.30)	90.60(84-98.80)	0.064
MCH, pg		29.90(27.40–32.30)	29.90(27.40–32.20)	30.40(27.40–33)	0.052
MCHC, g/L		331(319–342)	331(319–342)	331(319–341)	0.865
MPV, fL		9.90(9.20–10.60)	9.90(9.20–10.60)	9.80(9.30–10.60)	0.769
Glu, mmol/L		6.70(5.41-8)	6.70(5.40-8)	7.30(5.80–9.30)	< 0.001
PA, mg/L		168(101–235)	168(101–235)	169(123–226)	0.426
Glb, g/L		35.90(30.40–42.30)	35.90(30.40–42.40)	32.60(30.42–40.90)	0.657
LDH, U/L		228(180-313.65)	228(180–314)	224(185–296)	0.892
BASO, 10^9/L		0.02(0.01–0.03)	0.02(0.01–0.03)	0.02(0.01–0.03)	0.353
EOS, 10^9/L		0.10(0.02–0.24)	0.10(0.02–0.24)	0.10(0.02–0.23)	0.474
Fbg, g/L		3.02(1.84–4.11)	3.01(1.84–4.11)	3.10(2.12–4.26)	0.143
Hb, g/L		102(84–119)	102(83.31-118.63)	105(87.60–120)	0.103
D-Bil, µmol/L		2.89(1.75–5.31)	2.89(1.74–5.30)	2.88(1.89–5.50)	0.771
NEUT, 10^9/L		2.93(1.85–4.69)	2.93(1.84–4.69)	3.14(2.05–4.77)	0.110
TC, mmol/L		3.62(2.88–4.42)	3.62(2.87–4.42)	3.61(2.98–4.41)	0.468
T-Bil, µmol/L		6.90(4.60–11)	6.90(4.60-11.03)	7.14(4.63–10.46)	0.821
TP, g/L		68.90(62.40–75.40)	69(62.40–75.40)	68(62.30–74.60)	0.175

The continuous variables were expressed as median the median (IQR) after the normality distribution test. The categorical variables were expressed as number (percentage). ALB, albumin; WBC, white blood cell; APTT, activated partial prothrombin time; MONO, monocyte count; LDL-c, low density lipoprotein cholesterol; AMY, amylase; Ca, calcium; TG, triglyceride; HDL-c, high density lipoprotein cholesterol; GGT, γ-Glutamyl Transferase; ALT, alaninetransaminase; AST, aspartate aminotransferase; Cys-C, Cystatin C; RBC, red blood cell; RDW-CV, red blood cell distribution width CV; RDW-SD, red blood cell distribution width SD; HCT, hematocrit; Scr, serum creatinine; Ccr, creatinine clearance rate; CK, creatine kinase; K, kalium; I-Bil, indirect bilirubin; LYM, Lymphocyte count; Cl, chlorine; Mg, magnesium; Na, sodium; SUA, serum uric acid; TT, thrombin time; PT, prothrombin time; MCV, mean corpuscular volume; MCH, mean corpuscular hemoglobin; MCHC, mean corpuscular hemoglobin concentration; MPV, mean platelet volume; Glu, blood glucose; PA, prealbumin; Glb, globulin; LDH, lactate dehydrogenase; BASO, basophil count; EOS, eosinophil count; Fbg, fibrinogen; Hb, hemoglobin; D-Bil, direct bilirubin; NEUT, neutrophil count; TC, total cholesterol; T-Bil, total bilirubin; TP, total Protein

### Model performance and evaluation

**Fig. 2:**
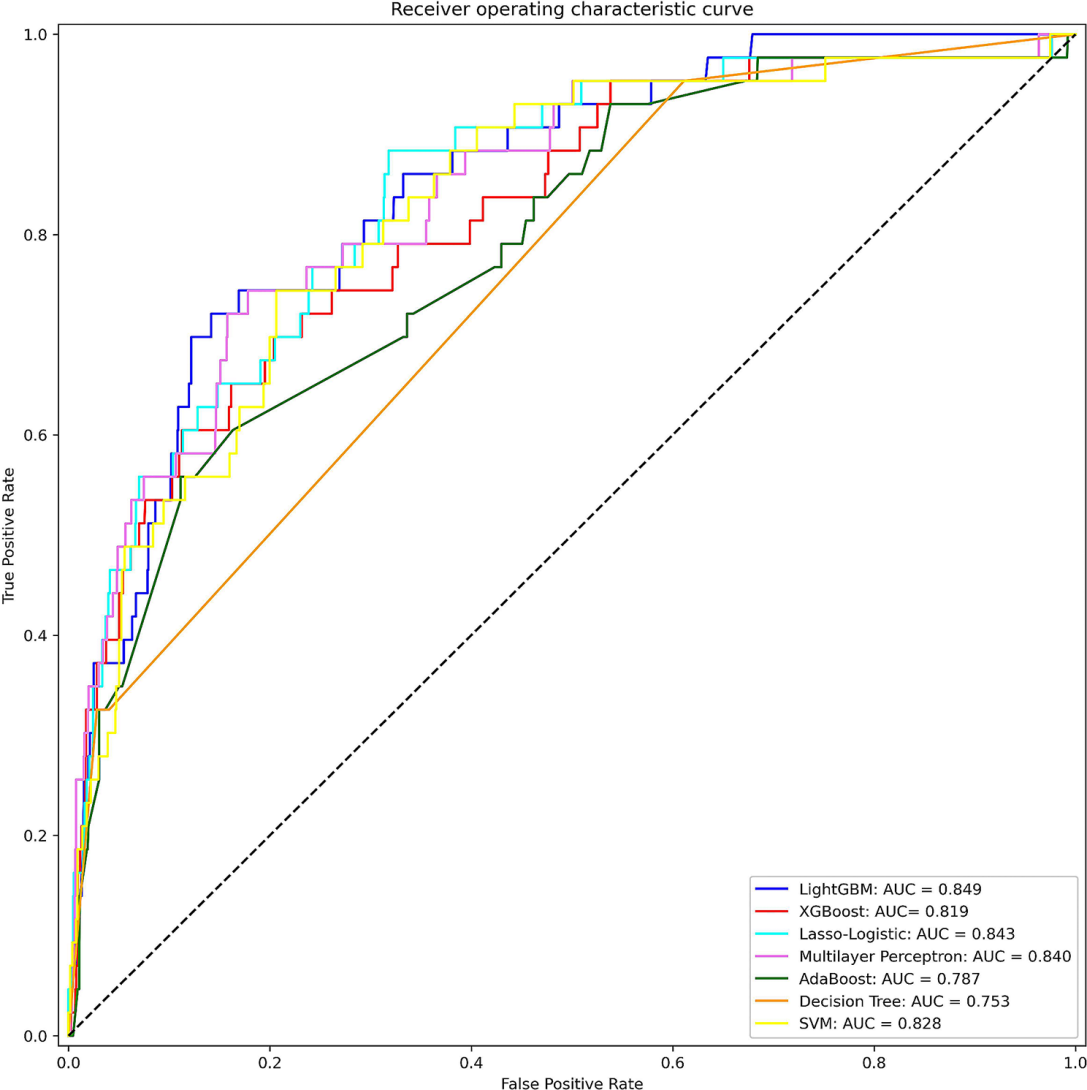
Assessment of the seven ML algorithms per the AUC of the ROC curve. AUC, area under the curve; ROC, receiver operating characteristic

The training set contained 5417 samples, whereas the validation set contained 1355 samples. Moreover, XGBoost, decision tree, AdaBoos, LightGBM, SVM, MLP, and lasso-logistic regression were built per the training set with the aforementioned 61 variables. Model performance evaluation was aided by the seven ML algorithms, as depicted in Table 2; Fig. 2. The LightGBM model demonstrated superior performance, achieving the highest AUC of 0.849 (95% CI, 0.814–0.883), whereas the decision tree exhibited the lowest AUC one (0.753; 95% CI, 0.704–0.803). Given that the LightGBM model exhibited the ideal performance across the four ML algorithms, it was deemed the best model.

**Table 2:** Performance of prediction models generated by the seven ML algorithms

Models	AUC	AUC 95% CI		Recall	SP	ACC	F1	PPV	NPV
		Lower bound	Upper bound						
LightGBM	0.849	0.814	0.883	0.721	0.858	0.757	0.238	0.143	0.989
XGBoost	0.819	0.779	0.859	0.698	0.796	0.784	0.176	0.101	0.988
AdaBoost	0.787	0.742	0.832	0.558	0.889	0.961	0.225	0.141	0.984
Multilayer Perceptron	0.840	0.804	0.876	0.744	0.822	0.967	0.207	0.120	0.990
Decision Tree	0.753	0.704	0.803	0.953	0.389	0.407	0.093	0.049	0.996
Support Vector Machine	0.828	0.790	0.866	0.744	0.793	0.675	0.185	0.106	0.990
Lasso-Logistic	0.843	0.807	0.878	0.884	0.682	0.744	0.153	0.084	0.994

### Explanation of risk factor

**Fig. 3:**
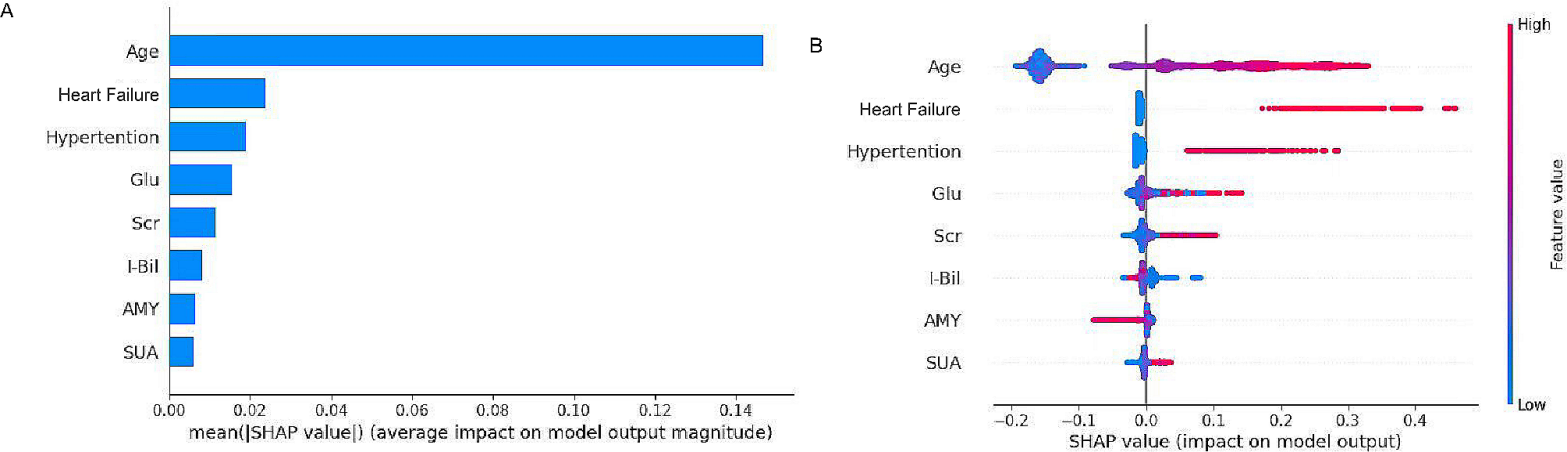
**A** an importance matrix plot of the LightGBM model depicting the significance of each variable in anticipating CHD risk in PLHIV. **B** SHAP summary plot of the top eight clinical attributes of the LightGBM model. Each point represents the SHAP value of a specific feature on a data point, indicating the magnitude and direction of that feature’s impact on the model’s predictive outcome. Red points denote high feature values with a positive incremental effect on the prediction; blue points denote low feature values with a negative decremental effect. Features are ranked from top to bottom by their average impact, highlighting their relative importance in the model’s decision-making process. Glu, glucose; Scr, serum creatinine; I-Bil, indirect bilirubin; AMY, amylase; SUA, serum uric acid

**Fig. 4:**
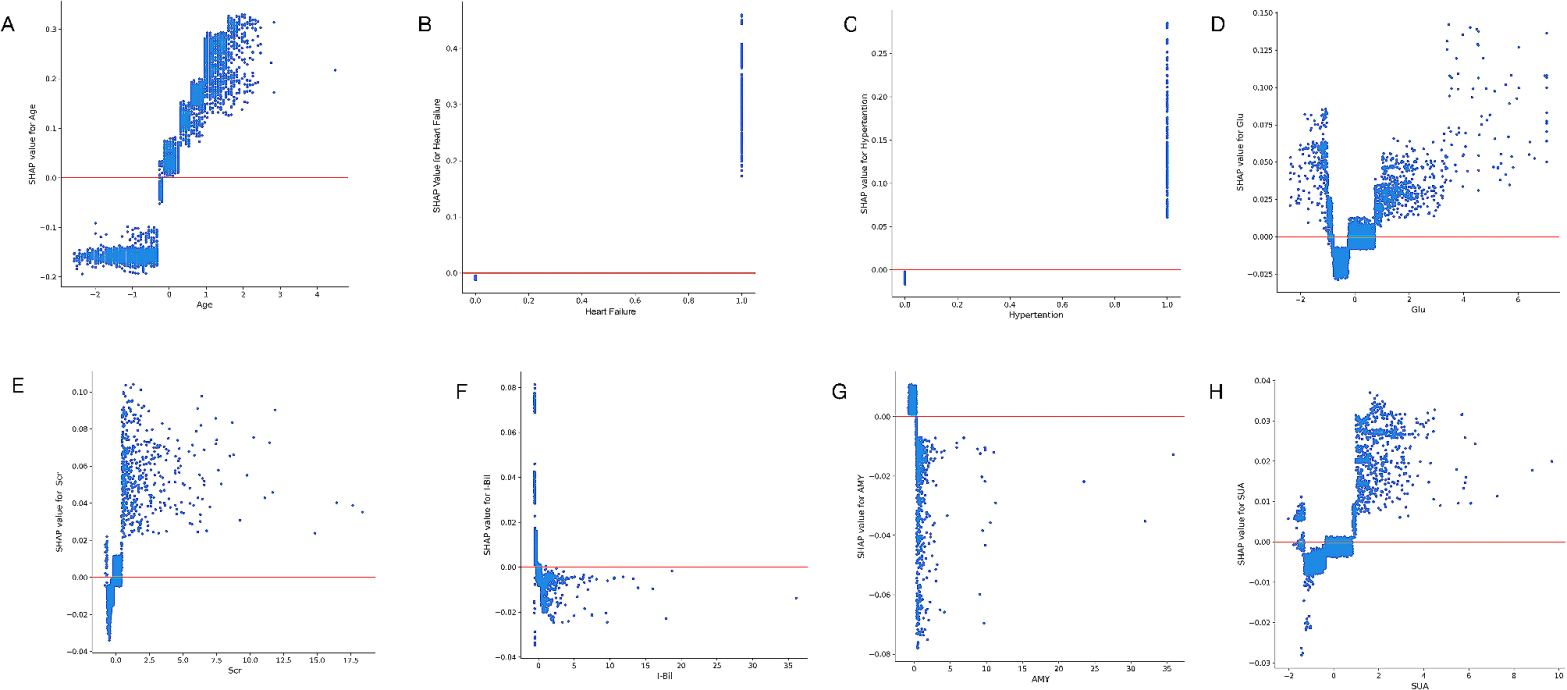
SHAP dependence plot of the LightGBM model, illustrating the influence of a single variable on the prediction. The blue points in the plot represent the SHAP values for this feature across different data points, with the horizontal position indicating the feature’s contribution to the predictive outcome. SHAP values greater than zero suggest an increased risk of CHD in PLHIV. The vertical axis represents the actual values of the feature, and the distribution of points reveals the relationship between the feature values and the risk of CHD. Glu, glucose; Scr, serum creatinine; I-Bil, indirect bilirubin; AMY, amylase; SUA, serum uric acid

SHAP aided the interpretation of the LightGBM model findings by computing the individual contribution of each variable. The importance matrix and SHAP summary plots for LightGBM are depicted in Fig. 3, whereas the SHAP dependence plot for the same is depicted in Fig. 4. Additionally, the importance matrix plot ranked variables contributing to CHD risk prediction among PLHIV from highest to lowest contribution per the baseline age, heart failure, hypertension, glucose (Glu), serum creatinine (Scr), indirect bilirubin (I-Bil), amylase (AMY), and serum uric acid (SUA) of the individuals (Fig. 3A). The SHAP summary (Fig. 3B) and SHAP dependence (Fig. 4) plots ascertained the influence of each variable on the CHD outcome.

As illustrated in the SHAP summary plot, higher feature values corresponded with a higher likelihood of CHD occurrence in PLHIV. The red and blue dots depicted higher and lower feature values, respectively. The high values of age, heart failure, hypertension, Glu, Scr, and SUA corresponded with a value of SHAP > 0, indicating that these features were vital risk factors for CHD in PLHIV. Generally, older PLHIV individuals (Fig. 4A) with heart failure and hypertension (Fig. 4B and C), high Glu (Fig. 4D), high Scr (Fig. 4E), poor I-Bil (Fig. 4F), low AMY(Fig. 4G), and high SUA (Fig. 4H) exhibited an elevated CHD risk.

### Applying the prediction model

**Fig. 5:**
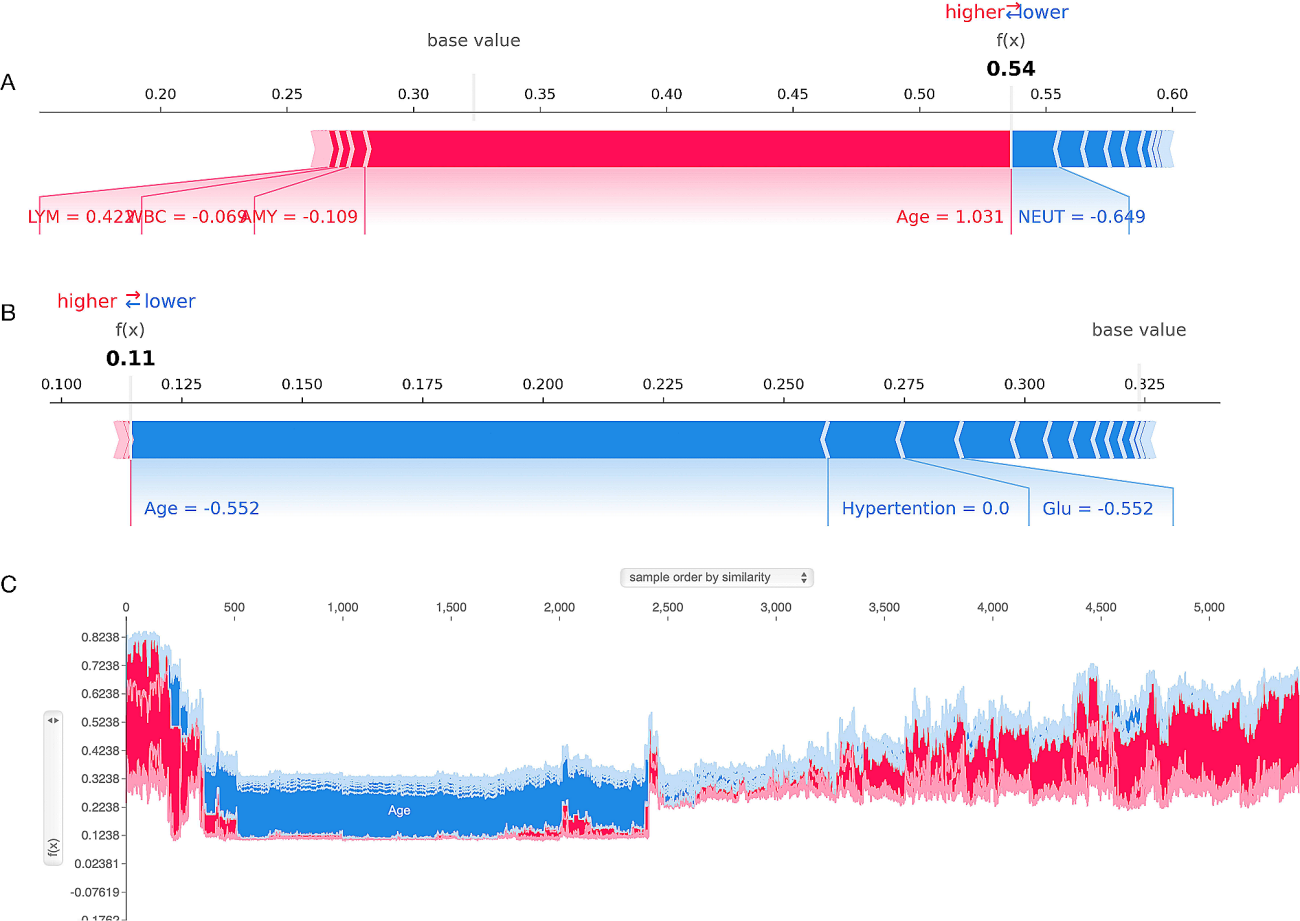
SHAP force plot for individuals in the dataset at high (**A**) or low (**B**) risk of CHD with PLHIV. C Values of SHAP (global interpretation) for the training set. The abscissa denotes each individual, and the ordinate depicts the value of SHAP. A greater appearance of red denotes a higher overall risk, conversely, a more pronounced blue indicates a comparatively lower risk of CHD

The actual application of the model is illustrated in Fig. 5. Red means the feature value elevates CHD probability, whereas blue denotes a reduction in CHD probability owing to the feature; *f(x)* represents the comprehensive value of SHAP for each individual. The base value depicts the mean value of SHAP for all samples. Hence, if *f(x)* was higher than the base value, the model would declare the individual as having CHD. Figure 5A illustrates that a PLHIV was accurately predicted to suffer from CHD, and Fig. 5B illustrates that a PLHIV without CHD was distinguished accurately. Therefore, the LightGBM model produced a sufficient distinction between CHD and non-CHD individuals and can denote different risk probabilities based on the individual circumstances of each patient. Figure 5C illustrates the values of SHAP predicted for each patient in the training set, with more red indicating a higher overall risk.

## Discussion

This study identified predictive risk factors for CHD among PLHIV and subsequently developed a CHD risk-prediction model using ML and easily retrieved clinical variables from EMR. Earlier studies have demonstrated that existing cardiovascular disease prediction models for HIV patients primarily include the D: A:D model (2010) for 5-year risk [36], the D: A:D model (2010) for 10-year risk [37, 38] the full D: A:D (2016) model for 5-year risk [39], the reduced D: A:D (2016) model for 5-years risk [39], the HIV MI-1 model [40] and the HIV MI-2 model [40]. These models are primarily based on Cox proportional risk models, Poisson regression models, and lasso and ridge regressions [41]. However, significant heterogeneity exists in the definition of cardiovascular disease among these models [41], and the prediction of CHD risk in HIV patients is susceptible to other confounding factors. To the best of our knowledge, this work is the first predictive model for HIV patient-specific CHD developed based on ML algorithms. Furthermore, we compared the predictive model performance of seven ML algorithms to establish the best model. Then, predictive model performance was compared across the seven ML algorithms. The observations revealed that the LightGBM model had the highest AUC, F1, and PPVs. Moreover, LightGBM is an effective implementation of the gradient-boosting learning algorithm, which is based on the decision-tree algorithm and uses n-lifting trees. It is superior to other algorithms in cases of prediction problems [42]. Furthermore, the algorithm is extensively used in regression and classification research with good detection results [42]. Accordingly, the SHAP method aided the explanation of the decision-making process adopted by the LightGBM algorithm and helped doctors intuitively understand its prediction process. The SHAP summary and dependency maps displayed heart failure, age, hypertension, Glu, I-Bil, Scr, AMY, and SUA to distinguish patients with HIV who were at low or high risk of CHD. Older PLHIV with high Glu, Scr, and SUA and with low I-Bil and AMY, combined with heart failure and hypertension, were at a higher risk of developing CHD. Additionally, both elevated glucose and hypertension were also risk factors for CHD in PLHIV, consistent with literature [8, 43].

In an investigation of the proteomic compositions of CD4 cells infected by HIV-1, Chan et al. [44] established that an elevation in fatty acid synthase (FASN) concentration post-infection and serum levels of inflammatory cytokines and insulin were positively correlated with FASN levels. This finding suggests that disrupting the lipid metabolism within HIV-infected cells of the immune system can cause systemic lipid metabolic disorders and inflammatory whole-body insulin resistance (IR), ultimately progressing to dysglycemia [45]. Additionally, the possible mechanisms of CHD include inflammation, endothelial-cell injury, thrombosis, oxidative stress, and glucose and lipid metabolism disorders [46]. Recent studies have demonstrated that IR contributes to coronary plaque formation and remodeling independent of traditional risk factors such as smoking, age, and hypertension [47]. Furthermore, certain studies have established that vascular stiffness is increased by endothelial-cell injury directly related to HIV infection or the activation of endothelial-cell proliferation by HIV proteins and cytokines, in association with ongoing hypertension-related endothelial damage. It may also contribute to the elevated incidence of CVD in individuals with HIV and hypertension [48].

Yue et al. [49] conducted a cohort study in Taiwan. They established robust relations between HIV infection and incident heart failure post-stratification of individuals by sex, age, and comorbidities. They also found that an HIV infection increases the risk of heart failure [50]. Growing evidence indicates that the severity of the HIV infection and the degree of HIV control may be key factors influencing heart failure risk [51]. In patients with CHD, coronary artery blood supply is insufficient, causing myocardial ischemia. Long-term ischemia cannot be effectively improved and in turn causes myocardial degeneration and even necrosis. It easily leads to complications, the most common being heart failure. Heart failure induces a decline in heart contractility and output reduction, thereby resulting in insufficient blood supply to important organs. This insufficient blood supply to the heart can further aggravate myocardial ischemia and even increase the severity of CHD.

This study also demonstrated that Scr, I-Bil, AMY, and SUA were risk factors for CHD in PLHIV. Bagheri et al. [52] explored the link between serum creatinine and the possibility and severity of CHD. They established that serum creatinine is significantly related to CHD. Meanwhile, other studies have revealed that creatinine levels in early HIV patients are higher than those in other groups [53]. Therefore, the Scr levels of PLHIV must be thoroughly monitored during hospitalization. These findings depicted that elevated I-Bil levels were protective for CHD with PLHIV. Marconi et al. [54]. conducted a veterans aging cohort study. The participants (regardless of HIV status) with elevated bilirubin levels are found to have a lower risk of incident total CVD, acute myocardial infarction, heart failure, and ischemic stroke events post-adjustment for known risk factors. Amylase also reportedly increases in individuals with acute HIV infection [55]. Hence, the serum amylase level of newly diagnosed PLHIV is related to CD4 cell count [56], i.e., CD4 cell count decreases with increased serum amylase.

Anti-retroviral therapy may also be the main cause of increased serum amylase in human immunodeficiency virus patients [56]. Park et al. [57] reported that coronary artery disease history (1.7, 1.01–2.87, *P* = 0.046) is related to heightened serum amylase or lipase when considering the prognosis, morbidity, and predisposition factors of individuals with elevated pancreatic enzyme levels post-cardiac arrest outside the institute. Serum amylase is a direct indicator of pancreatic injury [56], and studies have demonstrated that individuals with chronic pancreatitis have an elevated risk of atherosclerotic cardiovascular disease [58, 59]. Therefore, monitoring amylase alterations during PLHIV therapy positively influences the prevention or early detection of cardiovascular diseases.

Olaniyi et al. [60] established that uric acid content is significantly elevated in PLHIV relative to healthy controls. Uric acid, the end-product of purine metabolism in humans, is a cause of gout. However, it may also lead to the onset and progression of cardiovascular diseases, including atrial fibrillation, hypertension, chronic kidney disease, coronary artery disease, heart failure, and cardiovascular death. Thus, it can be used to predict cardiovascular prognosis [61]. Nicholson et al. [62] demonstrated that hyperuricemia and gout should be considered biomarkers of cardiovascular disease in PLHIV. Uric acid is elevated in PLHIV, and it contributes to cardiovascular disease onset and progression. Thus, alterations in blood uric acid levels should not be ignored when treating hospitalized PLHIV.

The present study had several strengths. For instance, it was real-world research pertaining to risk assessment utilizing 6772 samples, which was performed by comparing seven ML algorithms. The optimal prediction model, i.e., the LightGBM model, was found to have an improved generalizability advantage. It was a highly optimized GBDT algorithm that can incorporate several clinical variables. Furthermore, leveraging the benefits of an ML algorithm meant that the analysis can include various indicators, for example, kidney function, blood glucose, coagulation function, and liver function. Thus, it can aid the thorough assessment of the influencing factors. Furthermore, SHAP was a reliable technique to enhance the clinical interpretability of the LightGBM model output. Doctors can initiate reasonable referral recommendations and individualized CHD health-management suggestions to PLHIV.

This study also had several limitations. It was performed at a single institute, wherein the small sample and missing data derived from EMRs can produce a potential bias. Moreover, we focused on a single center, so only internal validation was conducted. External validation must be established using another dataset to demonstrate stability in the performance of the prediction model. Hence, more effort is needed to conduct multi-center prospective research with more opportunities for multi-center cooperation and better data-mining capabilities.

In summary, demographic and clinical variables were identified as predictive risk factors for CHD among PLHIV. Additionally, a CHD risk-prediction model was constructed for PLHIV using ML and EMR, which can support clinical management techniques for PLHIV in the EMR era.

## Data Availability

The datasets generated and/or analyzed are not publicly available owing to ethical and legal causes. Nevertheless, they can be made available from the corresponding author Jiannan Lv upon reasonable request.
